# Anomalous domain periodicity observed in ferroelectric PbTiO_3_ nanodots having 180° stripe domains

**DOI:** 10.1038/srep26644

**Published:** 2016-05-26

**Authors:** Jong Yeog Son, Seungwoo Song, Jung-Hoon Lee, Hyun Myung Jang

**Affiliations:** 1 Department of Applied Physics, College of Applied Science, Kyung Hee University, Suwon 446-701, Republic of Korea; 2 Department of Materials Science and Engineering, and Division of Advanced Materials Science, Pohang University of Science and Technology (POSTECH), Pohang 790-784, Republic of Korea; 3 Department of Physics, University of California, Berkeley, Berkeley, California 94720, USA

## Abstract

Nanometer-scale ferroelectric dots and tubes have received a great deal of attention owing to their potential applications to nonvolatile memories and multi-functional devices. As for the size effect of 180° stripe domains in ferroelectric thin films, there have been numerous reports on the thickness-dependent domain periodicity. All these studies have revealed that the domain periodicity (*w*) of 180° stripe domains scales with the film thickness (*d*) according to the classical Landau-Lifshitz-Kittel (LLK) scaling law (*w* ∝ *d*^*1/2*^) *d*own to the thickness of ~2 *nm*. In the case of PbTiO_3_ nanodots, however, we obtained a striking correlation that for the thickness less than a certain critical value, *d*_*c*_ (~35 *nm*), the domain width even increases with decreasing thickness of the nanodot, which surprisingly indicates a negative value in the LLK scaling-law exponent. On the basis of theoretical considerations of *d*_*c*_, we attributed this anomalous domain periodicity to the finite lateral-size effect of a ferroelectric nanodot with an additional effect possibly coming from the existence of a thin non-ferroelectric surface layer.

Ferroelectrics are receiving a great deal of attention because of their technological promise in leading toward miniaturized and efficient memory devices^1^. Like other ferroics, ferroelectrics are characterized by domain structures. Various forms of ferroelectric materials, such as ceramics, single crystals and thin films, exhibit a variety of different domain structures which include a stripe, mosaic, or vortex to minimize the total free energy which is composed of competing depolarization and domain-wall energy terms^2^,^3^,^4^,^5^,^6^,^7^,^8^,^9^,^10^. Ferroic domains that are ordered along one unique direction but with opposite polarity or magnetic moment are called 180° domains. It has long been known that the width of 180° stripe magnetic domains closely follows the so-called Landau-Lifshitz-Kittel (LLK) scaling law^11^. This law was later extended to ferroelectric domains by Mitsui and Furuichi^2^. According to the scaling law, the domain width (*w*) is directly proportional to the square root of the crystal thickness (*d*), namely, *w* = *Ad*^*γ*^, where *A* is a proportionality constant and *γ* is the scaling-law exponent (=1/2).

Extensive theoretical studies^3^,^12^,^13^,^14^,^15^,^16^ have been carried out to examine the validity of the LLK scaling law on ferroelectric multiple domains, in conjunction with a variety of experimental studies done by employing various methods that include x-ray scattering^5^,^6^, piezoelectric force microscopy^10^, and scanning transmission electron microscopy^12^. Until now, all the experimental studies for 180° stripe domains reveal that the LLK scaling law with the exponent around 1/2 is valid down to the thickness of ~2 *nm*^5^,^14^,^15^. However, there appears one interesting study done by Catalan *et al.*^10^. According to their study, the domain size of multiferroic BiFeO_3_ thin films having irregular domain walls is noticeably larger than those of other ferroelectrics having the same thickness and the observed scaling-law exponent (*γ*) of 0.59 deviates quite substantially from its normal value of 1/2. They correlated the former with a strong magneto-electric coupling at domain walls while attributing the latter to a fractal-like Hausdorff dimension^10^.

Among numerous ferroelectrics, lead titanate (PbTiO_3_; PTO hereafter) has been most extensively studied and is known as a prototype of displacive ferroelectrics without exhibiting any over-damping of the resonance-type soft phonons^17^,^18^. Currently, PTO-based nano-scale dots and tubes have received a great deal of attention owing to their potential applications to high-density nonvolatile memories and multi-functional devices^19^,^20^. As for the size effect of 180° stripe domains in PTO thin films, there have been numerous reports on the thickness-dependent domain periodicity^5^,^6^,^13^,^14^,^15^. All these studies reveal that the domain periodicity of 180° stripe domains scales with the film thickness (*d*) according to the classical LLK scaling law down to the thickness of ~2 *nm*^5^,^14^,^15^.

Contrary to the experimentally observed LLK scaling behavior, a theoretical solution of the Laplace equation for rigorously evaluating the depolarizing-field energy of a normal ferroelectric suggests that the LLK scaling law does break for the thickness less than a certain critical size, *d*_*c*_ :^21^ the domain width (*w*) even increases with decreasing thickness below *d*_*c*_. Unlike 180° stripe domains, the experimental *w* of the (101)-type 71° domains in rhombohedral BiFeO_3_ indeed follows this anomalous behavior with *d*_*c*_ as large as ~200 nm^22^. Huang *et al.*^22^ were able to qualitatively account for the observed thickness-dependent domain width for the (101)-type 71° domains by theoretically considering the sum of the elastic and domain-wall energies in (101) boundary^22^. Similarly, the domain width of a pseudo-proper ferroelectric is predicted to be inversely proportional to the film thickness (*i.e.*, *w* ~ *λ*^2^/*d*) for a very thin film^23^, where *d* ≪ *λ* and *λ* is the characteristic length associated with the linear coupling between the polarization (secondary order parameter) and the primary order parameter (*e.g*., *λ* = ~15 *nm* for TbMnO_3_)^23^.

In view of the above discrepancy between the theoretical prediction^21^,^23^ and the experimental observations^5^,^14^,^15^, it is of great scientific importance to experimentally clarify whether there exists any critical thickness (*d*_*c*_) for the validity of the LLK scaling law or not. Until now, however, the LLK scaling law for 180° stripe ferroelectric domains has been experimentally tested using thin films^5^,^6^,^10^,^12^,^13^,^14^,^15^ where the lateral dimension (*L*) is practically infinite. Considering this, we have critically examined the effect of the lateral dimension on the validity of the LLK scaling law using ferroelectric PTO nanodots having a variety of different lateral sizes.

Herein we present a quite striking experimental result that the thickness-dependent domain width of the PTO nanodot having 180° stripe domains does not obey the LLK scaling law but is characterized by a negative exponent (*γ* < 0) below a certain critical thickness, *d*_*c*_. On the basis of theoretical considerations of *d*_*c*_, we have come to the conclusion that the finite lateral-size effect, in addition to the possible existence of a ferroelectrically inactive surface layer, should be taken into account to properly explain the difference in the scaling behavior between ferroelectric films and dots.

## Experimental Results

**Figure 1:**
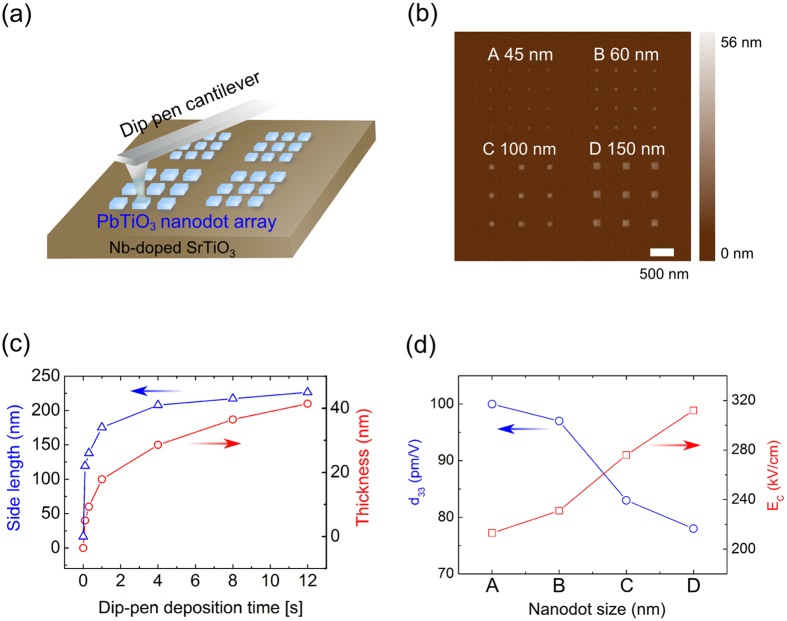
PbTiO_3_ nanodots on a Nb-doped SrTiO_3_ substrate. (**a**) Dip-pen nanolithography (DPN) of PbTiO_3_ nanodots. (**b**) An AFM image for PbTiO_3_ nanodot array having four different size classes. The array was formed by the DPN method. (**c**) Side length and thickness of PbTiO_3_ nanodots as a function of the dip-pen deposition time. (**d**) *d*_*33*_ value and the coercive electric field (*E*_*c*_) plotted as a function of the nanodot size, where A, B, C, and D indicate the dot size in (**b**).

A ferroelectric PTO nanodot array was fabricated on an Nb-doped SrTiO_3_ (STO) substrate using the dip-pen nanolithography (DPN) method [Fig. 1(a)]. This method had been successfully applied to the fabrication of PTO nanodots having a variety of different lateral sizes^20^. Figure 1(b) shows atomic force microscopy (AFM) images of PTO nanodots with several different size classes. These DPN-formed nanodots are rectangle-shaped, indicating a high degree of crystallinity. From the high-resolution transmission electron microscopy image, we confirmed that these PTO nanodots are characterized by the tetragonal *4* *mm* (*C*_*4v*_) symmetry with the polar *c*-axis perpendicular to the substrate plane^20^. The dimension (thickness, side length) of the nanodot can be controlled by adjusting the dip-pen deposition time [Fig. 1(c)]. For a short deposition time (<1 sec), the lateral dimension (side length *L*) increases rapidly while the thickness (*d*) increases rather steadily with increasing deposition time. As shown in Fig. 1(d), *d*_*33*_ value decreases while the coercive electric field increases with the lateral size, which presumably reflects size-dependent depolarization effects^20^.

**Figure 2:**
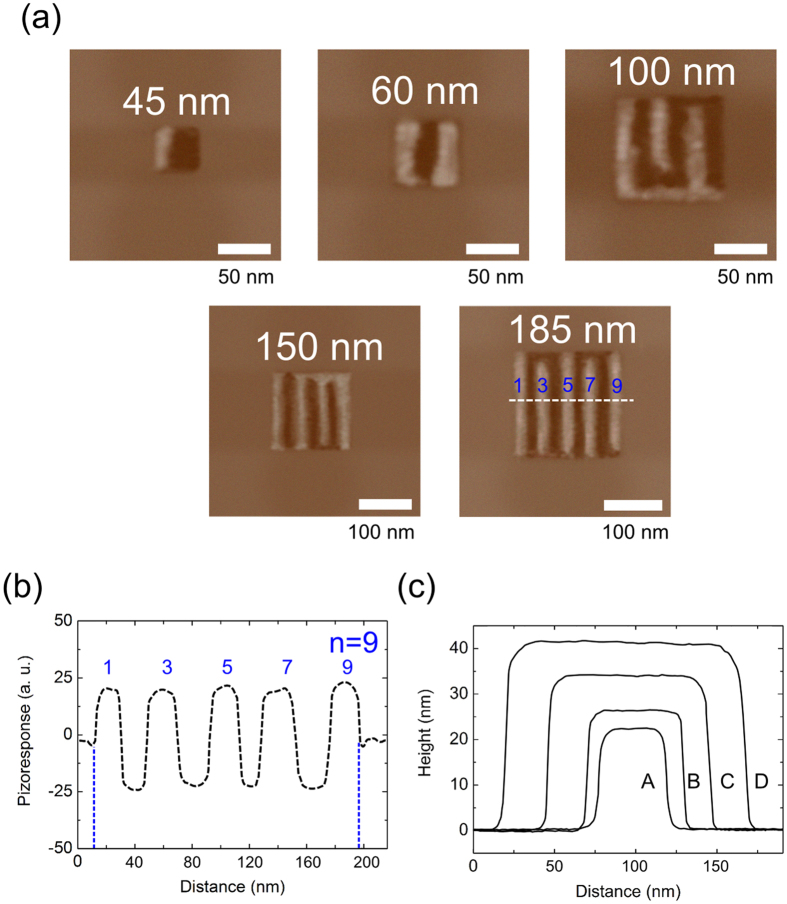
PbTiO_3_ nanodots having 180° stripe domains. (**a**) PFM images of the five selected nanodots with different lateral sizes. (**b**) A PFM line profile of the PbTiO_3_ nanodot having a lateral dimension of 185 nm as an example. (**c**) AFM line profiles of the four selected nanodots with different lateral sizes, where A, B, C, and D denote the lateral size (*i.e*., side length) of 45, 60, 100, and 150 nm, respectively.

To examine the effect of the lateral dimension (*L*) on the validity of the LLK scaling law, PFM (piezoelectric force microscopy) measurements were carried out for a series of PTO nanodots having a variety of different lateral sizes ranging from 45 nm to 500 nm. Figure 2(a) presents PFM images of the five selected PTO nanodots with different lateral sizes. The PFM images indicate that regardless of the lateral dimension, the PTO nanodots grown on an Nb-doped STO substrate are characterized by ferroelectric 180° stripe domains. The PFM line profile of the PTO nanodot having a lateral dimension of 185 nm [∵ (197-12 nm)] is shown in Fig. 2(b) as an example. This line profile demonstrates that the nanodot is composed of nine 180° stripe domains. Figure 2(c) presents the AFM line profiles of the four selected nanodots [A, B, C, and D in Fig. 1(b)] showing that the thickness (*d*) of the nanodot increases with the lateral dimension (*L*). In the below, we summarize the four characteristic geometric parameters of the five 180° stripe domains presented in Fig. 2(a) in the ascending order of the dot thickness, *d*: (i) **dot A** (*L* = 45 nm, *n* = 2, *d* = 21.5 nm, *w* = 22.5 nm), (ii) **dot B** (*L* = 60 nm, *n* = 3, *d* = 25 nm, *w* = 20.0 nm), (iii) **dot C** (*L* = 100 nm, *n* = 6, *d* = 35 nm, *w* = 16.7 nm), (iv) **dot E** (*L* = 185 nm, *n* = 9, *d* = 40.7 nm, *w* = 20.6 nm), and (v) **dot D** (*L* = 150 nm, *n* = 7, *d* = 42 nm, *w* = 21.4 nm), where the AFM line profile of the **dot E** having *L* = 185 nm is not presented in Fig. 2(c) since its height is very close to that of the **dot D**, and *n* denotes the number of 180° stripe domains in a given nanodot. Thus, *L = nw*. Similar to the present nanodots, 180° stripe-domain structures were also observed in epitaxially grown PTO films (on STO substrates) for the film thickness down to ~2 nm^5^.

**Figure 3:**
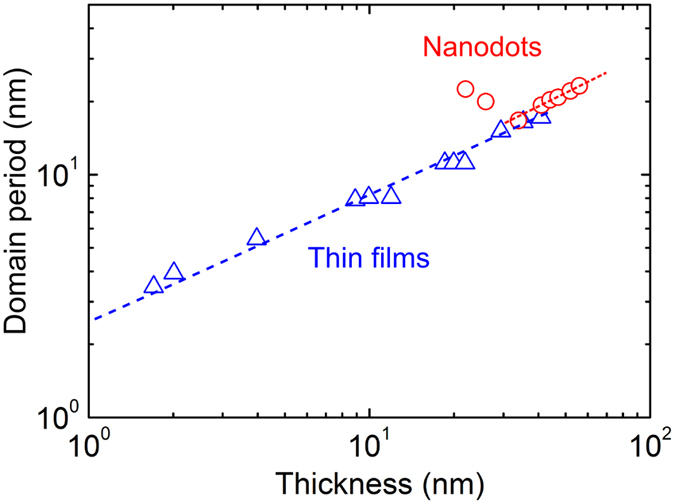
The thickness-dependent domain periodicity (*w*) of the PbTiO_3_ nanodot is compared with that of the epitaxially grown PbTiO_3_ film. Data for the thin films (blue color triangles) were taken from the published result^5^.

We now focus on the correlation between the domain width (*w*) and the dot thickness (*d*). In the case of PTO thin films, there is a good linear correlation between log *w* and log *d* with the scaling exponent (*λ*) of 1/2 [Fig. 3], which closely follows the LLK scaling law^5^. In contrast, the PTO nanodots exhibit a quite striking correlation. For *d* > 35 nm, the PTO nanodots also follow the scaling law with the estimated exponent of 0.52. However, the domain width even increases with decreasing thickness for *d* < 35 nm [Fig. 3]. This kind of surprising results has never been observed in ferroelectric thin films.

## Theoretical Analysis and Discussion

### Gibbs free-energy function of a ferroelectric nanodot

To clarify the main cause of the observed striking result in the PTO nanodots, we have theoretically considered the effect of the lateral dimension on the difference in the scaling behavior between a nanodot and a thin film and consequently examined the possibility of occurrence of the anomalous domain periodicity for the dot thickness less than a certain critical value (*d*_*c*_). For this purpose, we first formulated the Gibbs free-energy of a ferroelectric nanodot (having a finite lateral dimension) as a function of the domain width and obtained a modified LLK scaling law that accounts for, at least qualitatively, the observed anomalous domain periodicity for *d* < 35 nm.

**Figure 4:**
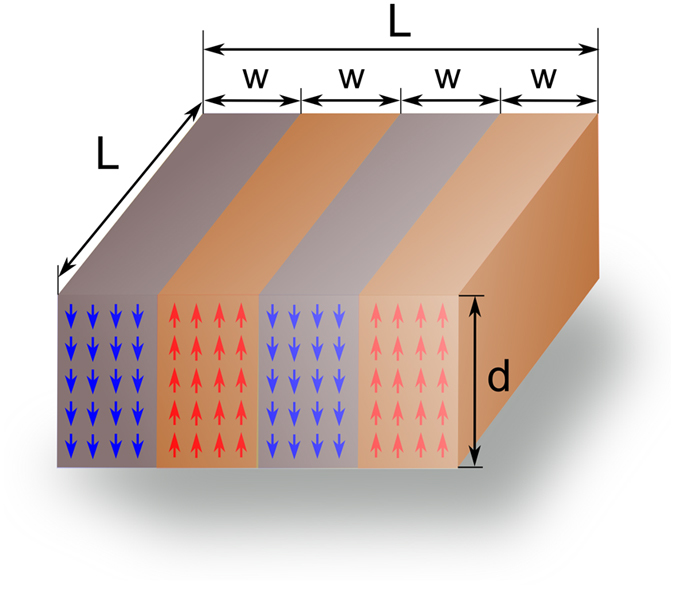
A schematic representation of 180^o^ stripe domains in a given PbTiO_3_ nanodot, where *L*, *w*, and *d* are described in the text.

Let us consider a ferroelectric nanodot (*i.e.*, nano-rectangle) having a dimension of *L* × *L* × *d* with the domain width of *w*, as schematically depicted in Fig. 4. Then, the Gibbs free-energy function of a ferroelectric nanodot (having the volume of *L*^2^*d*) with respect to that of a paraelectric nanodot (at given *T* and *P*) can be written in terms of *w*, *d*, and *L* as


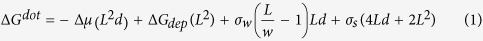


where Δ*μ* denotes the difference in the bulk free energy per unit volume between the paraelectric and ferroelectric phases at given *T* and *P*, namely, 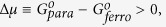
and Δ*G*_*dep*_ designates the depolarization-field energy per unit area. On the other hand, *σ*_*w*_ in Eq. (1) denotes the domain-wall energy per unit area whereas *σ*_*s*_ represents the surface tension of four side faces of a retangular nanodot having the area of *L* × *d* per face. Here *σ*_*s*_ can be viewed as the excess surface free energy (per unit area) of the ferroelectric rectangle [*σ*(*T*, *P*)] with respect to the surface tension of the paraelectric rectangle (*σ*_*p*_) having the same dimension, *i.e.*, *σ*_*s*_ ≡ *σ*(*T*, *P*)−*σ*_*p*_. Thus, the last term takes care of the excess surace free energy of a nano-rectangle having six mutually orthogonal faces. For mathematical simplicity, we assume that 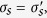 where 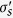 denotes the excess surface free energy of top (or bottom) surface of a retangular nanodot. It can be shown, however, that this simplification does not alter our interpretation of the anomalous domain periodicity. In addition, it is theoretically shown that 180° stripe domains under an external electric field behave as elastic domains due to the converse piezoelectric effect^24^. Under this condition, the interatomic elastic-interaction between 180° ferroelectric domains is an important factor in determining the effective dielectric and piezoelectric responses of thin film constrained by a substrate^24^. Since we are examining the domain periodicity in the absence of an external electric field, we neglect this interatomic elastic-interaction term in our theoretical analysis.

According to the Landau-Ginzburg theory, the difference in the free energy between ferroelectric and paraelectric states can be written in terms of *P* (polarization order-parameter) and its gradient as


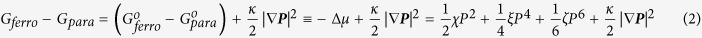


where *κ* denotes the Ginzburg gradient-energy coefficient. In Eq. (2)*χ* (below the Curie temperature) and *ξ* are negative while *ζ* is positive for a displacive ferroelectric that undergoes a discontinuous first-order phase transition^25^. From Eq. (2), one can deduce the following expression for the equilibrium bulk polarization 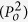 under the condition of zero gradient:


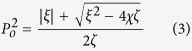


Substituting Eq. (3) into Eq. (2) yields the following expression that relates Δ*μ* with the Landau expansion coefficients (*i.e.,* dielectric stiffness coefficients):


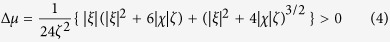


Considering Eq. (2), one can evaluate the domain-wall energy, *σ*_*w*_, by carrying out the following integration if the polarization profile across the domain wall, *P*(*x*), is established:


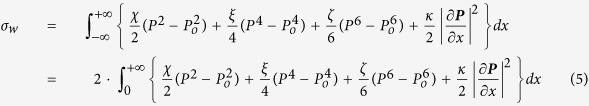


The second expression of Eq. (5) is valid for a symmetrical domain boundary.

Let us now return to Eq. (1). Substituting *nw* for *L* (Fig. 4) and dividing Δ*G*^*dot*^ by *L*^*2*^, one obtains the following expression for the Gibbs free-energy function of a ferroelectric nanodot per unit area (Δ*G*):


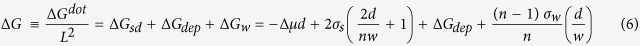


where the first two terms in the right-hand-side of Eq. (6) represent the free energy of the single-domain state per unit area (Δ*G*_*sd*_) whereas the last term denotes the domain-wall energy (Δ*G*_*w*_) of a nano-rectangle having the dimension of *L* × *L* × *d*. Δ*G*_*dep*_ in Eq. (6) represents the depolarization-field energy per unit area. Let us now define the following parameter for future convenience:


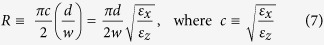


*ε*_*z*_ in Eq. (7) denotes the relative dielectric permittivity along the unique polarization axis, *i.e*., *ε*_*c*_ for PTO. Then, Δ*G*_*w*_ can be rewritten in terms of *R* and *c* as


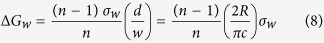


Similarly, Δ*G*_*sd*_ can be rewritten using *R* and *c* as


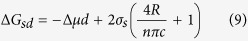


On the other hand, the following complicated expression of Δ*G*_*dep*_ can be obtained by solving the Laplace equation under suitable continuity conditions for the electric field (***E***) and the dielectric displacement vector (***D***):^21^


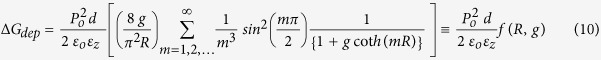


where *g* is defined by *g* ≡ (*ε*_*x*_*ε*_*z*_) = *cε*_*z*_. The term, 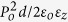, represents the energy of a plate condenser per unit area having thickness *d*, filled by a dielectric with the permittivity *ε*_*z*_ and carrying surface charges ± *P*_*o*_. On the other hand, *f*(*R*, *g*) is a dimensionless function which is equal to the terms inside the parenthesis of Eq. (10).

According to Kopal, Bahnik, and Fousek^21^, there exists a certain critical film thickness above which the electrostatic interaction of the domain surfaces can be neglected. This thickness is given by


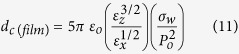


For *d* > *d*_*c*_, Δ*G*_*dep*_ can be simplified by the following well known expression^2^,^14^,^21^,^26^, instead of the complicated expression presented in Eq. (10):


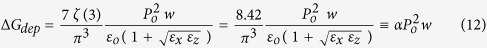


where *ζ*(3) is the Riemann zeta function and is equal to 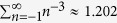.

### A modified scaling law for ferroelectric nanodots

Under the condition of *d* > *d*_*c*_ (*i.e*., “thick” plates approximation), Eq. (6) can be written explicitly using Eq. (12) as


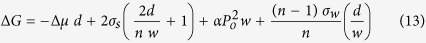


Then, one can obtain the following expression for the equilibrium domain width of a ferroelectric rectangle (dot) by setting (*∂*Δ*G/∂w*)_*d*_ = 0:


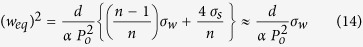


The last expression of Eq. (14) represents an asymptotic scaling law under the condition of *n* → ∞ and, thus, corresponds to a thin film having an infinite lateral dimension. As expected, this asymptotic law correctly reproduces the classical *w*^2^−*d* scaling law. In contrast, the first expression indicates that the *w*^2^−*d* scaling law may not be valid for a nanodot where *n* is a small integer and depends on *d*.

One cannot neglect the mutual electrostatic interaction between the domain (plate) surfaces for the dot thickness smaller than *d*_*c*_. In this case, Eq. (12) should not be used to evaluate Δ*G*_*dep*_. Instead, one can derive a modified scaling law by exploiting Eqs (8), (9), and (10) and subsequently by setting (∂Δ*G*/∂*R*)_*g*_ = 0. To do this, let us first evaluate (∂Δ*G*_*dep*_/∂*R*)_*g*_. In doing this, one has to consider the following two obvious relations: *sin*^2^(*mπ*/2) = 1 for *m* = 1, 3, 5.… (odd integers) and *sin*^2^(*mπ*/2) = 0 for *m* = 2, 4, 6.… (even integers). Incorporating this result into Eq. (10), one obtains the following expression of (∂Δ*G*_*dep*_/∂*R*)_*g*_:


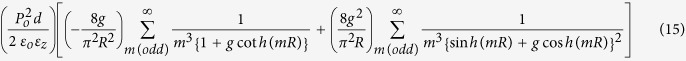


As defined in Eq. (7), *R* is proportional to (*d/w*). Substituting Eq. (15) into the requirement that 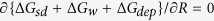, one can eventually obtain the following non-classical relation between *w* and *d* for *d < d*_*c*_:


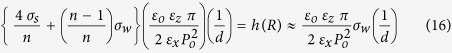


where


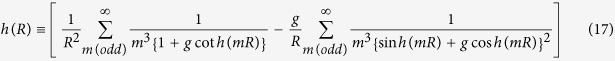


Eq. (16) clearly indicates that the simple *w*^2^−*d* scaling law is no more valid for *d* < *d*_*c*_. According to Eq. (16), the domain width even increases with decreasing *d*, which successfully accounts for the observed anomalous domain periodicity (Fig. 3) for *d* < 35 nm (*d*_*c*_). It is interesting to note that the last expression of Eq. (16) which is asymptotically valid for *n* → ∞ (infinite lateral dimension) exactly coincides with the modified scaling equation for thin films as proposed by Kopal *et al.*^21^.

### Effect of the lateral dimension on the critical thickness

Though the anomalous behavior of the domain periodicity (for *d* < *d*_*c*_) in the PTO nanodots can be qualitatively explained by adopting Eq. (16), we still have one important question to be resolved in the case of thin films: Why does the classical *w*^2^−*d* scaling law describe the domain periodicity well down to the thickness of ~2 *nm*? In other words, why is the critical thickness (*d*_*c*_) not observed down to ~2 *nm* in the case of thin films? To answer this question, we have considered the most prominent difference between a nanodot and a thin film, which is the lateral dimension (*L*), thus, the number of domains, *n*.

In the case of thin films where the lateral dimension (*L*) is practically infinite, the critical thickness (*d*_*c*_) for neglecting the electrostatic interaction of the domain surfaces is given by Eq. (11). According to Eq. (11), the critical film thickness can be rewritten as 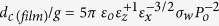. Since the right-hand side of this relation is constant for fixed values of *ε*_*z*_, *ε*_*x*_, *σ*_*w*_, and *P*_*o*_, the critical film thickness is proportional to 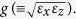 For *σ*_*w*_ = 5 × 10^−3^ *J•m*^*−2*^, *P*_*o*_ = 0.2 *C•m*^*−2*^, and *c* = 5, *d*_*c*(*film*)_ is approximately given by *g* ×2.2 × 10^−13^ (*m*). Combining this result with the observation that *d*_*c(film)*_ <2 *nm* one can deduce that *g* ≤ ~1 × 10^+ ^13. Numerical calculations of the thickness-dependent (*w*/*g*) using Eqs (16) and (17) further indicate that regardless of the value of *g*-parameter used, the domain width (*w*) increases rapidly with decreasing film thickness (*d*_*film*_) for *d*_*film*_ < *d*_*c*_.

We will then deduce the corresponding expression of the critical thickness for a ferroelectric nanodot (*d*_*c*(*dot*)_) where *L* or *n* is finite. To do this, we first assume that the domain width at *d*_*c*_ is proportional to *d*_*c*(*dot*)_ itself. Thus, one can establish that *w*_*c*(*dot*)_ = *kd*_*c*(*dot*)_, where *k* is a proportionality constant. By exploiting this proportionality and Eq. (14) in the vicinity of *d*_*c*_, one can eliminate *w*_*c*(*dot*)_ from this relation and obtain the following expression for *d*_*c*(*dot*)_:


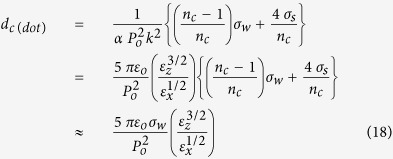


where *n*_*c*_ denotes the number of distinct domains in the nanodot having the critical thickness of *d*_*c*(*dot*)_. *k*^*2*^-term appeared in the first expression of the above equation was eliminated in the second expression by comparing Eq. (11) with the first expression in the asymptotic thin-film limit where *n* → ∞. It is worth noting that the last expression of Eq. (18), which corresponds to the asymptotic thin-film limit, does coincide with Eq. (11) which had been deduced by Kopal and co-workers for thin films^21^.

Comparing the second expression of Eq. (18) with Eq. (11), one obtains the following ratio of the critical thickness for a nanodot having *n* distinct 180° domains (*d*_*c*(*dot*)_) to that for a thin-film having an infinite lateral dimension (*d*_*c*(*film*)_):


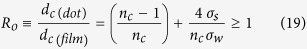


This equation predicts that the asymptotic value of the above ratio in the limit of *n*_*c*_ → ∞ (*i.e*., thin film) is 1 as expected. One can qualitatively estimate *d*_*c*(*film*)_ by taking a suitable value of (*σ*_*s*_/*σ*_*w*_). Assuming (*σ*_*s*_/*σ*_*w*_) ≈ 5 and plugging *n*_*c*_ = 6 and *d*_*c*(*dot*)_ ≈ 35 *nm* into Eq. (19), one predicts that *d*_*c*(*film*)_ = 8.4 *nm* which is in direct disagreement with the observation that *d*_*c*(*film*)_ ≤  2 *nm*^5^,^14^,^15^. Herein, *n*_*c*_and *d*_*c*(*dot*)_ values were taken from those of the **dot C** (see “Experimental Results” section). One can immediately obtain the following expressions from Eq. (19):


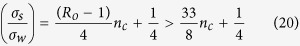


**Figure 5:**
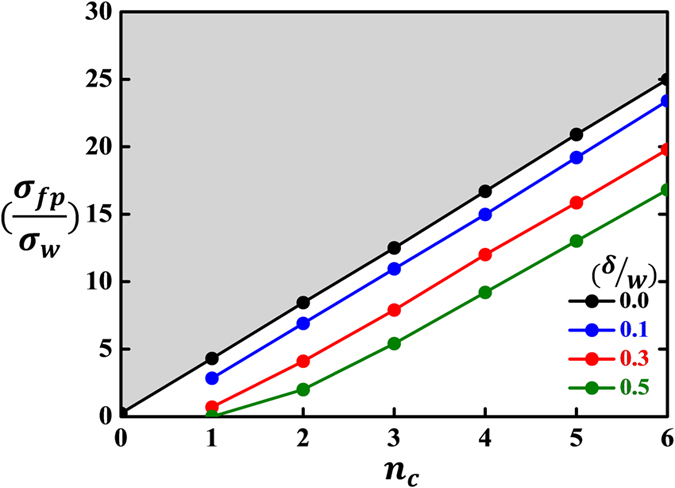
The minimum allowed value of (*σ*_*fp*_/*σ*_*w*_) plotted as a function of *n*_*c*_. As shown in the figure, the minimum allowed value depends sensitively on the thickness of a non-ferroelectric surface layer, *δ.* Among the four lines, the black line which corresponds to (*δ*/*w*) = 0 denotes the minimum allowed value of (*σ*_*s*_/*σ*_*w*_) in the absence of a thin non-ferroelectric surface layer. The shaded region represents the allowed region of *n*_*c*_-dependent (*σ*_*s*_/*σ*_*w*_) for *δ* = 0.

In obtaining the last expression of Eq. (20), we used the observation that *d*_*c*(*dot*)_ ≈ 35 nm (Fig. 3) and *d*_*c*(*film*)_ <2 *nm*^5^,^14^,^15^. This indicates that the ratio of *σ*_*s*_ to *σ*_*w*_ should be greater than a certain critical value which, in turn, is linearly proportional to *n*_*c*_ with the intercept being 1/4. The allowed region of *n*_*c*_-dependent (*σ*_*s*_/*σ*_*w*_) is shown in Fig. 5 using a shaded mark. According to this theoretical prediction, the minimum value of (*σ*_*s*_/*σ*_*w*_) that satisfies the above inequality for *n*_*c*_ = 6 is 25.0 which seems to be unrealistically high to be accepted. This suggests that the critical thickness, *d*_*c*_, is actually determined not only by the lateral-size effect but also by some other factor. In other words, the lateral-size effect alone cannot account for the observation that *R*_*o*_ ≡ *d*_*c(dot)*_/*d*_*c(film)*_ >17.5(=35/2).

### Possible existence of a non-ferroelectric surface layer

It is now clear that the lateral-size effect alone cannot satisfactorily explain the observed difference in the *w*^*2*^*-d* scaling behavior between a film and a nanodot [Fig. 3]. To resolve this puzzling situation, we postulate the presence of a ferroelectrically inactive thin surface layer. This postulation is supported by the experimental observations of thin non-ferroelectric surface layers in perovskite-based ferroelectrics such as BaTiO_3_^27^,^28^,^29^. According to the experimental estimate by transmission electron microscopy, the thickness of this surface layer is around 10 *nm*^28^. First-principles calculations^30^,^31^ and phase-field simulations^32^ also support the existence of a surface relaxation layer. The size-dependent depolarization effects on *d*_*33*_ and *E*_*c*_, as shown in Fig. 1(d), also suggest a non-ferroelectric surface layer. The most important effect of this postulation is considered to be the compensation of the depolarizing field^33^ by the lateral surfaces of a nanodot.

We have examined the possible existence of a non-ferroelectric surface layer and its effect on the optimized surface structure. For this purpose, we performed first-principles density-functional theory (DFT) calculations on the basis of the generalized gradient approximation (GGA) method implemented with the projector augmented wave (PAW)^34^ pseudopotential using the Vienna *ab initio* Simulation Package (VASP)^35^. All of the DFT calculations were performed using the plane wave cutoff energy of 500 eV. We have considered the stacking of PbTiO_3_ along [001], which consists of alternating TiO_2_ and PbO layers. For actual calculations, we adopted slabs of 11 atomic layers of the in-plane polarized *c*(2 × 2) surface unit cell for the PbO termination^31^. For polarized films (within in-plane ferroelectric (FE) distortion), we found that in-plane FE and anti-ferrodistortive (AFD) distortions are concurrently enhanced at the PbO-terminated surface region, which leads to the formation of a structural surface phase with coexisting [100]-oriented FE and AFD distortions. However, this surface phase does not possess any FE polarization component along the principal [001] polar direction.

Having examined the possible existence of a surface phase by *ab initio* DFT calculations, we have thermodynamically considered this issue by suitably modifying the Gibbs free-energy function of a ferroelectric nanodot having surface layers^36^. We eventually obtained the following approximation for the critical-thickness ratio in the presence of a non-ferroelectric surface layer with the thickness *δ*:^36^


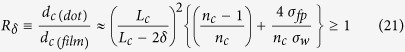


where *L*_*c*_ is the lateral dimension of a nanodot at the critical dot-thickness, *R*_*δ*_ denotes the ratio of the two critical thicknesses in the presence of a non-ferroelectric surface layer of the thickness *δ*. *σ*_*fp*_ appeared in Eq. (21) denotes the interfacial tension between the ferroelectric nanodot and the paraelectric surface layer. The most prominent difference between Eq. (19) and Eq. (21) is the introduction of a geometry term, {*L*_*c*_/(*L*_*c*_−2*δ*)}^2^, in Eq. (21), which always enhances *R*_*δ*_. More importantly, Eq. (21) tells us that both the existence of a non-ferroelectric surface layer of the thickness *δ* and the finite lateral-size effect determine *R*_*δ*_, thus *d*_*c*(*dot*)_. Assuming (*σ*_*fp*_/σ_*w*_) = 10, *δ* ≈ 15 nm and plugging *n*_*c*_ = 6, *L*_*c*_ ≈ 100 *nm* into Eq. (21), one obtains *d*_*c*(*film*)_ of ~2.3 *nm* which qualitatively agrees with the observation that *d*_*c*(*film*)_ ≤ ~2 *nm*.

Similar to the derivation of Eq. (20), one can obtain the following expression of (*σ*_*fp*_/*σ*_*w*_) directly from Eq. (21):


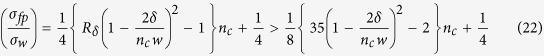


Again, we used the observation that *d*_*c*(*dot*)_ ≈ 35 nm (Fig. 3) and *d*_*c(film)*_ <2 *nm* in obtaining the last expression of Eq. (22). Unlike Eq. (20), however, there is no linear correlation between (*σ*_*fp*_/*σ*_*w*_) and *n*_*c*_. Notice that the right-hand side of Eq. (22) exactly coincides with that of Eq. (20) when *δ* = 0. The boundary between the allowed and prohibited regions of the *n*_*c*_-dependent (*σ*_*fp*_/*σ*_*w*_) is plotted in Fig. 5 for four selected values of (*δ*/*w*) including *0*. The minimum allowed value of (*σ*_*fp*_/*σ*_*w*_) decreases significantly with increasing value of *δ* at a given *n*_*c*_, suggesting that the formation of a thin non-ferroelectric surface layer substantially reduces the minimum allowed value of (*σ*_*fp*_/*σ*_*w*_). However, our derivation of Eq. (21) adopts a couple of unjustified assumptions^36^. We thus leave this problem (*i.e*., a rigorous theoretical treatment of the LLK scaling law for a nanodot having a non-ferroelectric surface layer) as a future challenging task. On the basis of all these results, we have come to the conclusion that the finite lateral-size effect (*n*), in addition to the possible existence of a ferroelectrically inactive surface layer, should be taken into account to properly explain the difference in the scaling behavior between ferroelectric films and nanodots.

## Conclusion

For the dot thickness larger than the critical value (*d*_*c*_) of ~35 nm, the width of 180° stripe domains scales with the thickness of the PTO nanodot according to the classical LLK scaling law. For the dot thickness smaller than *d*_*c*_, however, we obtain a quite striking correlation that the thickness-dependent domain width is experimentally represented by a negative exponent. On the basis of theoretical considerations of *d*_*c*_, we attribute this anomalous domain periodicity to the finite lateral-size effect of a ferroelectric nanodot with an additional effect possibly coming from the existence of a non-ferroelectric surface layer. *Ab initio* DFT calculations support the existence of a structural surface phase but with the absence of a FE polarization component along the principal [001] polar direction.

## Experimental Methods

Dip-pen nanolithography (DPN) method was used to fabricate a ferroelectric PTO nanodot array^20^. DPN, which enables us to form nanopatterns with various molecules at desired places, is one of noble atomic force microscopy (AFM) techniques that utilize both electric force microscopy (EFM) and piezo-response force microscopy (PFM). Unlike DPN involving polymers, we adopted sequential drying and annealing processes for the fabrication of PTO nanodots. We used a PbO-excess precursor sol modified with a low-viscosity alcohol. We first formed PTO-precursor sol on an Nb-doped SrTiO_3_ (STO) substrate. To elaborate the DPN process, we used an Nb-doped STO substrate that had an atomically flat surface with well-aligned terraces formed by HF treatment and annealing. After the deposition, we slowly dried these precursor sol nanodots at room temperature. For the crystallization of ferroelectric perovskite PTO, we annealed these nanodots under an oxygen atmosphere at 650 °C for 1 min by rapid thermal annealing. The size of the PTO nanodots was controlled by suitably adjusting the dip-pen deposition time. Piezoelectric hysteresis loops were measured by employing piezoelectric force microscopy (PFM) at a frequency of 10 kHz. PFM images of the PTO nanodots were observed using a high-resolution electric force mode of the PFM, where a platinum-coated Si_3_N_4_ cantilever tip was employed.

## Additional Information

**How to cite this article**: Son, J. Y. *et al.* Anomalous domain periodicity observed in ferroelectric PbTiO_3_ nanodots having 180° stripe domains. *Sci. Rep.* **6**, 26644; doi: 10.1038/srep26644 (2016).
